# Preoperative endoscopic versus percutaneous transhepatic biliary drainage in potentially resectable perihilar cholangiocarcinoma (DRAINAGE trial): design and rationale of a randomized controlled trial

**DOI:** 10.1186/s12876-015-0251-0

**Published:** 2015-02-14

**Authors:** Jimme K Wiggers, Robert JS Coelen, Erik AJ Rauws, Otto M van Delden, Casper HJ van Eijck, Jeroen de Jonge, Robert J Porte, Carlijn I Buis, Cornelis HC Dejong, I Quintus Molenaar, Marc GH Besselink, Olivier RC Busch, Marcel GW Dijkgraaf, Thomas M van Gulik

**Affiliations:** 1 Department of Surgery, Academic Medical Center, Meibergdreef 9, Room IWO-A1.119, 1105 AZ Amsterdam, The Netherlands; 2 Department of Gastroenterology, Academic Medical Center, Amsterdam, The Netherlands; 3 Department of Radiology, Academic Medical Center, Amsterdam, The Netherlands; 4 Clinical Research Unit, Academic Medical Center, Amsterdam, The Netherlands; 5 Department of Surgery, Erasmus Medical Center, Rotterdam, the Netherlands; 6 Department of Surgery, University of Groningen, University Medical Center Groningen, Groningen, the Netherlands; 7 Department of Surgery, Maastricht University Medical Center and NUTRIM School for Translational Research in Metabolism, Maastricht, the Netherlands; 8 Department of Surgery, University Medical Center Utrecht, Utrecht, the Netherlands

**Keywords:** Perihilar cholangiocarcinoma, Resection, Preoperative biliary drainage, Complications

## Abstract

**Background:**

Liver surgery in perihilar cholangiocarcinoma (PHC) is associated with high postoperative morbidity because the tumor typically causes biliary obstruction. Preoperative biliary drainage is used to create a safer environment prior to liver surgery, but biliary drainage may be harmful when severe drainage-related complications deteriorate the patients’ condition or increase the risk of postoperative morbidity. Biliary drainage can cause cholangitis/cholecystitis, pancreatitis, hemorrhage, portal vein thrombosis, bowel wall perforation, or dehydration. Two methods of preoperative biliary drainage are mostly applied: endoscopic biliary drainage, which is currently used in most regional centers before referring patients for surgical treatment, and percutaneous transhepatic biliary drainage. Both methods are associated with severe drainage-related complications, but two small retrospective series found a lower incidence in the number of preoperative complications after percutaneous drainage compared to endoscopic drainage (18-25% versus 38-60%, respectively). The present study randomizes patients with potentially resectable PHC and biliary obstruction between preoperative endoscopic or percutaneous transhepatic biliary drainage.

**Methods/Design:**

The study is a multi-center trial with an “all-comers” design, randomizing patients between endoscopic or percutaneous transhepatic biliary drainage. All patients selected to potentially undergo a major liver resection for presumed PHC are eligible for inclusion in the study provided that the biliary system in the future liver remnant is obstructed (even if they underwent previous inadequate endoscopic drainage). Primary outcome measure is the total number of severe preoperative complications between randomization and exploratory laparotomy. The study is designed to detect superiority of percutaneous drainage: a provisional sample size of 106 patients is required to detect a relative decrease of 50% in the number of severe preoperative complications (alpha = 0.95; beta = 0.8). Interim analysis after inclusion of 53 patients (50%) will provide the definitive sample size. Secondary outcome measures encompass the success of biliary drainage, quality of life, and postoperative morbidity and mortality.

**Discussion:**

The DRAINAGE trial is designed to identify a difference in the number of severe drainage-related complications after endoscopic and percutaneous transhepatic biliary drainage in patients selected to undergo a major liver resection for perihilar cholangiocarcinoma.

**Trial registration:**

Netherlands Trial Register [NTR4243, 11 October 2013].

**Electronic supplementary material:**

The online version of this article (doi:10.1186/s12876-015-0251-0) contains supplementary material, which is available to authorized users.

## Background

Perihilar cholangiocarcinoma (PHC) is a biliary malignancy originating at or near the hepatic duct confluence that typically causes biliary obstruction with concomitant cholestasis [1]. Surgical resection of PHC, using a combined extrahepatic bile duct resection and partial liver resection, offers the best chance for long-term survival with a reported median survival of 19–39 months in large series [2]. However, liver surgery in cholestatic patients with PHC is associated with high risks of postoperative morbidity and mortality, as reported up to 68% and 18%, respectively [3,4].

Preoperative biliary drainage is used to create a safer environment for liver surgery in PHC [5]. It reduces jaundice, improves nutritional status and liver function, reduces bacterial translocation, and improves the ability of the liver to regenerate postoperatively [6]. Owing to these effects, preoperative biliary drainage has been shown to reduce postoperative mortality, especially in extended liver resections [7]. Nonetheless, biliary drainage can be harmful when drainage related complications further deteriorate the patients’ condition or increase the risk of postoperative morbidity [8-10]. These complications potentially outweigh the benefits of biliary drainage, and should be minimized in order to optimize surgical outcomes.

The two most commonly used drainage techniques are endoscopic biliary drainage (EBD) or percutaneous transhepatic biliary drainage (PTBD). EBD is often used to treat symptoms of jaundice in regional centers before referral to specialty centers for surgical management. However, preoperative EBD in PHC is associated with a high rate of complications such as cholangitis and pancreatitis. As an alternative, PTBD reportedly has a high rate of technical success and theoretically, is associated with a lower incidence of cholangitis because there is no retrograde bacterial contamination from the gut. Nonetheless, other drawbacks have been reported of PTBD including hemorrhage, catheter dislocation, and patient discomfort. Only two small retrospective series (n = 68 and n = 101) have compared the incidence of preoperative complications, consistently reporting fewer complications after PTBD compared to EBD (18-25% versus 38-60%, respectively) [11,12]. These studies were obviously flawed by their retrospective study design and heterogeneity in procedure-site (i.e. regional versus specialty center). Prospective studies are needed to confirm the potential benefit of preoperative PTBD compared to EBD.

The present study randomizes patients between preoperative EBD and PTBD. Patients are eligible for inclusion when they have presumed PHC on imaging, when they are selected to potentially undergo a major liver resection, and when they have biliary obstruction in the future liver remnant (FLR). The study is based on an “all-comers” design, so patients may also be included if they require additional preoperative biliary drainage after previous inadequate EBD in a regional center before referral. The primary outcome measure of the study focuses on the total number of severe preoperative drainage-related complications.

## Methods/Design

### Study objectives

The aim of this study is to compare EBD and PTBD in terms of the total number of preoperative severe drainage-related complications (primary outcome), the success of biliary drainage, quality of life, and postoperative morbidity and mortality.

### Design

The present study is a multicenter randomized controlled superiority trial. Patients are randomized to preoperative EBD or PTBD by minimization using computer-generated allocation (www.tenalea.net). The randomization procedure is stratified for three factors, including: (attempted) biliary drainage procedures before referral to the enrolling center (drainage naïve versus drainage non-naive); the level of bile duct involvement (Bismuth type 1, 2, 3A or 3B versus type 4); and enrolling center.

### Participating centers

Five specialty centers for PHC treatment in the Netherlands are currently enrolling patients, including the Academic Medical Center in Amsterdam, the Erasmus Medical Center in Rotterdam, the University Medical Center Groningen, the Maastricht University Medical Center, and the University Medical Center Utrecht.

### Ethics

This study is conducted in accordance with the principles of the Declaration of Helsinki and the Dutch Medical Research Involving Human Subjects Act. The medical ethical committee of the Academic Medical Center in Amsterdam approved the protocol on September 2, 2013. An addendum to the protocol was approved March 5, 2014. Secondary approval was sought from all local ethics committees. Informed consent is obtained from each participating patient in oral and written form prior to randomization. The DRAINAGE trial is registered in the Dutch Trial Register with identification number NTR4243.

### Study population

The study is based on an “all-comers” design. All patients with presumed PHC, who are selected by the multidisciplinary team meeting at one of the enrolling centers to potentially undergo a major liver resection, are eligible for inclusion in the study when they have biliary obstruction in the FLR.

#### Inclusion criteria

According to the 7th edition of the American Joint Committee on Cancer staging, PHC is defined as a tumor originating in the common hepatic duct, the hepatic duct confluence, or in the left or right hepatic duct. [1]. The latter category may also include patients with an intrahepatic tumor that invades the hepatic duct confluence. Pathologic confirmation of PHC is not required prior to inclusion in the study. Patients are selected to potentially undergo a major liver resection if preoperative imaging with CT and/or MRCP shows a resectable tumor without evidence of distant metastases (including lymph node metastases beyond the hepatoduodenal ligament) [1]. Portal vein resection and reconstruction may be required to obtain tumor-free resection margins.

Biliary obstruction in the FLR is separately defined for patients with or without previous EBD procedures (Table 1). For drainage naïve patients, it is defined as a total bilirubin level of at least 50 μmol/L. For patients with previous EBD procedures, it is defined as a persistently rising total bilirubin level above 50 μmol/L (i.e. no stent placed or insufficiently draining stent) or as persistent biliary dilatation in the FLR on imaging (i.e. previous stent placed in contralateral side of liver).

**Table 1:** **Definitions of biliary obstruction in the future liver remnant**

Patient type	Definition of inadequate biliary drainage
Drainage naïve	Serum Bilirubin >50 μmol/L.
Drainage non-naïve	Persistently rising total bilirubin level above 50 μmol/L (i.e. no stent placed or insufficient draining stent).
	Persistent biliary dilatation in the FLR on imaging (i.e. previous stent placed in contralateral side of liver).

Patients are ‘drainage non-naïve’ if they underwent previous (attempted) EBD before referral to one of the enrolling centers.

#### Exclusion criteria

Since the primary outcome measure focuses on complications, patients are not eligible for inclusion if they have incompletely recovered from any side effect of previous biliary drainage procedures. Patients are required to be off antibiotic treatment for at least 5 days. Other exclusion criteria include any contra-indication for major liver surgery (e.g. ECOG/WHO score ≥3), technical contra-indication for either EBD or PTBD (e.g. previous gastrojejunostomy), or refusal to provide informed consent.

### Primary outcome measure

The primary outcome is the total number of severe drainage-related complications measured between randomization and exploratory laparotomy. The study is designed to detect superiority of PTBD. Some patients lose eligibility to undergo major liver resection before exploratory laparotomy (e.g. distant metastases found at staging laparoscopy, deterioration of the patients’ condition, or diagnosis of benign disease precluding the need for surgical therapy). For these patients, the number of drainage-related complications is measured until 7 days after the decision to cancel exploratory laparotomy, or until 90 days after randomization (whichever comes first).

A severe complication is defined as any complication related to the drainage procedures, leading to an additional invasive intervention, (extended) hospitalization, or death. Definitions for potential severe drainage-related complications, as evaluated in previous studies, [11,13] are shown in Table 2.

**Table 2:** **Definitions of severe complications in the primary outcome measure**

Severe complication	Criteria
Cholangitis	Elevation in temperature more than 38,5°C and Leukocytes ≥10 *10^9^/L, thought to have a biliary cause, without concomitant evidence of acute cholecystitis, requiring invasive intervention.
Acute cholecystitis	Radiologic evidence of cholecystitis, elevation in temperature more than 38.5°C and Leukocytes ≥10*10^9^/L, and requirement of percutaneous drainage or emergency cholecystectomy.
Stent/ catheter dysfunction	Rising bilirubin level after therapeutic success had initially been obtained, without signs of cholangitis or cholecystitis, requiring new cannulation of the tumor.
Acute pancreatitis	Abdominal pain and a serum concentration of pancreatic enzymes (amylase or lipase) ≥3 times the upper limit of normal, that requires ≥1 one night of hospitalization.
Hemorrhage	Clinical evidence of bleeding with the need of a blood transfusion.
Perforation	Retroperitoneal or bowel-wall perforation documented by any radiographic technique requiring intervention.
Portal vein thrombosis	Clinical evidence of thrombosis confirmed on colour Doppler US as absence of flow compatible with occlusion, precluding liver surgery.
Dehydration	Severe dehydration with electrolyte disturbances resulting from excessive fluid loss through externally draining catheters, requiring rehydration in the clinical setting.

To exclude bias in determining events pertaining to the primary outcome measure, a blinded adjudication committee will review all events and evaluate whether events count as severe complication according to the proposed definitions.

### Secondary outcome measures

Secondary outcome measures will be used to select the preferred method if there is no difference in the primary outcome at conclusion of the study. Secondary outcome measures include:

- The separate incidence of preoperative cholangitis;
- The number of drainage procedures required to achieve technical success;
- The proportion of patients with therapeutic success at 7 days after technical success;
- The total number of drainage procedures that involved (attempted) stent (re-) placement;
- The proportion of patients requiring crossover treatment;
- Interval bilirubin decrease at 7 days and 14 days after biliary drainage relative to the bilirubin level at randomization;
- The number of patients with rescheduled or cancelled laparotomy due to severe drainage-related complications;
- Quality of life, measured with the EORTC Quality of Life Questionnaire C-30 (QLQ-C30) and its biliary cancer module (QLQ-BIL21);
- Postoperative morbidity and mortality, as defined in Table 3, among patients who underwent combined extrahepatic bile duct and major liver resection. The International Study Group of Liver Surgery (ISGLS) definitions for post-hepatectomy liver failure, bile leakage and hemorrhage are used [14-16].

**Table 3:** **Definitions of postoperative morbidity and mortality**

Event	Criteria
**Postoperative mortality**	Any reason of death within 90 days after major liver resection.
**Postoperative morbidity**	
Posthepatectomy liver failure	Increasing INR and bilirubin on or after postoperative day 5 plus deviation from regular clinical management (Grade B definition according to the International Study Group of Liver Surgery [ISGLS]) [14].
Cholangitis	Elevation in temperature more than 38.5°C and Leukocytes ≥10*10^9^/L, thought to have a biliary cause, without concomitant evidence of acute cholecystitis, requiring invasive intervention [13].
Hepaticojejunostomy (biliary) leakage	Drainage of fluid with an increased bilirubin level three times greater than the serum level on or after postoperative day three; or the need for interventions as the result of bile collections or biliary peritonitis; or direct visual evidence of defect at anastomoses (definition according to ISGLS) [15].
Intra-abdominal abscess formation	Intra-abdominal fluid collection with positive cultures identified by ultrasonography or computed tomography, associated with persistent fever and elevations of white blood cells [13].
Wound infection	Requiring intervention; otherwise considered as minor complication [13].
Portal vein thrombosis	Conclusive radiologic evidence of thrombosis [13].
Hemorrhage	A drop in haemoglobin level >3 g/dl post-operatively compared with the post-operative baseline level and/or post-operative transfusion of ≥2 units packed red blood cells for a falling haemoglobin and/or the need for radiological intervention (such as embolization) and/or re-laparotomy to stop bleeding (Grade B/C haemorrhage according to ISGLS) [16].
Emergency re-laparotomy	Any (other) reason following major liver resection [13].
Pneumonia	Pulmonary infection with radiological confirmation and requiring antibiotic treatment [13].

### Study outline

**Figure 1:**
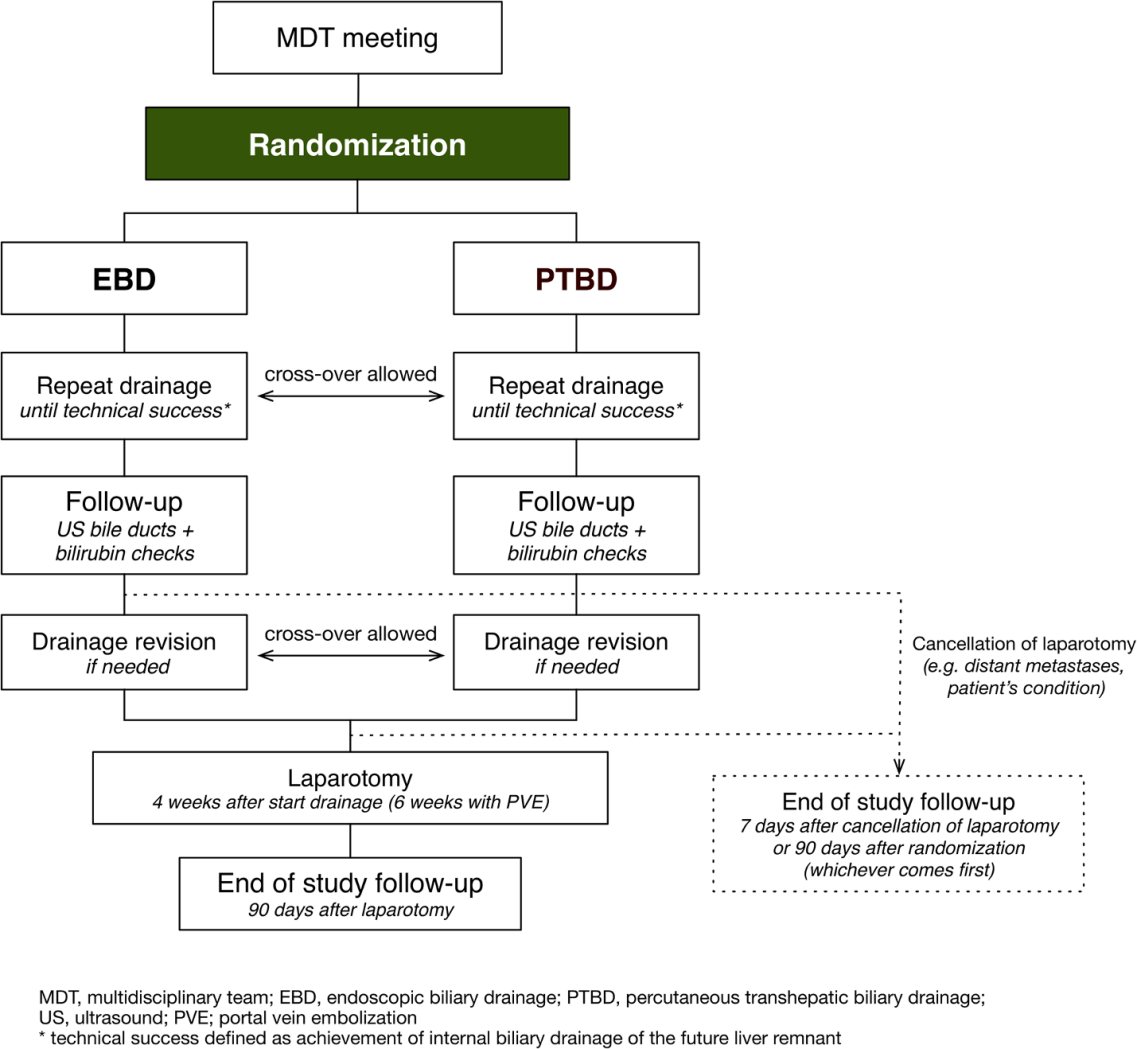
**Flowchart of the study.**

The general study outline is presented in Figure 1. All patients with presumed PHC presenting at the participating centers are evaluated in a multidisciplinary team meeting. Patients are eligible for inclusion if all in-and exclusion criteria are met according to the multidisciplinary team meeting based on clinical history and CT and/or MRCP imaging. Next, the type of liver resection indicated is determined in the multidisciplinary team meeting, defining the side of the FLR and primary aim of preoperative biliary drainage. Planned liver resections may consist of right (extended) hemihepatectomy, left extended hemihepatectomy, or left hemihepatectomy: the corresponding FLR that should be drained preoperatively consists of the left liver segments, the right posterior segments, or the right anterior + posterior segments.

After informed consent has been obtained, patients are randomized to undergo EBD or PTBD of the FLR within 5 days. The allocated drainage procedure should be repeated until internal biliary drainage of the FLR has been achieved (i.e. technical success). Follow-up is done at 7 days after technical success: therapeutic success at follow-up is defined as normal caliber bile ducts in the FLR on ultrasonography and a minimum 20% decrease in the total bilirubin level compared to the reference level at randomization. Additional total bilirubin assessments are performed at 14 days after technical success, and at admission for exploratory laparotomy, to evaluate patency of the biliary drainage. Revisional drainage procedures may be required if complications occur after biliary drainage, as described in Table 2. Complications are treated according to local guidelines. Crossover treatment is allowed if the allocated drainage procedure is considered to be no longer technically feasible (e.g. cholangitis with multiple isolated segments after EBD, requiring additional PTBD).

Additional staging (e.g. percutaneous biopsy or staging laparoscopy) may be used to further determine resectability of the tumor after randomization and initial biliary drainage. Patients who loose eligibility to undergo major liver resection because distant metastases are found at additional staging remain included in the intention-to-treat analysis, as described above.

Exploratory laparotomy is intentionally scheduled at 4 weeks after randomization, or at 6 weeks after randomization if the patient requires portal vein embolization. A radical resection is pursued at exploratory laparotomy if the diagnosis of resectable PHC without distant metastases is confirmed. Resections consist of excision of the liver hilum en bloc with (extended) hemihepatectomy including the caudate lobe, and complete lymphadenectomy of the hepatoduodenal ligament. Portal vein excision and reconstruction are used when necessary. If present, transhepatic percutaneous drains are left in-situ and used as transanastomotic drains to facilitate healing of the hepaticojejunostomies.

### Preoperative biliary drainage

Technical success of biliary drainage is defined as successful stent/catheter placement providing internal biliary drainage of the future liver remnant. Preoperative biliary drainage preferably consists of unilateral drainage of the FLR, but bilateral drainage may be indicated as evaluated by the treating physician (e.g. need for portal vein embolization or bilateral cholangitis) [17].

#### Endoscopic biliary drainage

A sphincterotomy with blended electrosurgical current may be used to facilitate the insertion of multiple stents and/or brush cytology. Opacification upstream of the obstruction is intentionally restricted to segments that are aimed to be drained during the procedure. Bile duct(s) unintentionally opacified upstream from the obstruction should be drained during the same procedure. Polyethylene endoprostheses (size 10Fr) are then pushed in position over the catheter. Directly after the procedure, a 100 mg Diclofenac suppository is administered to the patient for the prevention of post ERCP-pancreatitis [18].

#### Percutaneous transhepatic biliary drainage

The technique of PTBD involves the use of ultrasound guidance, a thin Chiba needle and a 0.014-inch guidewire to gain access to the biliary system. Antegrade cholangiography will be used to localize the site of obstruction. The guidewire is manoeuvred through the stenosis. A catheter is placed with its distal end in the duodenum for internal-external-drainage. Externally draining bile is collected approximately during the first 48 h, after which the catheter is closed in order to achieve internal drainage.

#### Antibiotic treatment

Standard antibiotic prophylaxes are administered at the start of each biliary drainage procedure, according to local protocol as described in an additional file [see Additional file 1]. Prophylactic antibiotic protocols vary between centers, but they are similar for EBD and PTBD procedures in the same center.

### Data collection and monitoring

All preoperative and postoperative complications will be monitored. Patients complete questionnaires (QLQ-C30/BIL21) at the day of randomization, and at 7 days, 28 days, and 90 days after randomization. There will be regular contact between the trial coordinator and the participating centers. Baseline and outcome data are collected by designated data managers using a standardized case record form [see Additional file 2], which is monitored by the trial coordinator.

### Statistical analysis

The DRAINAGE trial is a superiority trial, designed to detect a reduction in the primary outcome measure in favor of PTBD. The analysis will be based on the intention-to-treat principle.

#### Sample size

Determination of the sample size is split in a provisional and definitive calculation, because only little retrospective evidence is currently available for power estimation. A previous study from the primary study center showed a severe complication rate of 60% in the EBD group versus 18% in the PTBD group. In the DRAINAGE trial, a complication rate of 50% is expected after EBD and 25% after PTBD (i.e. a relative decrease of 50%). This estimation was further expanded with proportions for the number of complications, as shown in Table 4. Accounting for a 3% dropout, 53 patients are needed in each arm resulting in a provisional required sample size of 106 patients (Wilcoxon/Mann–Whitney rank-sum test for ordered categories; α = 0.95; β = 0.8).

**Table 4:** **The estimated number of complications in each study group used to calculate the provisional sample size**

	EBD	PTBD
0 complications	50%	75%
1 complication	25%	17%
≥2 complications	25%	8%
Total proportion	100%	100%

The estimated proportion of patients is presented for each categorical number of complications.

Interim analysis after inclusion of 53 patients (50%) will provide the definitive sample size, which will be calculated based on the observed number of complications in the EBD group. The definitive sample size will be determined similar to the provisional sample size, again using a relative decrease of 50% in the proportion of complications unless the absolute decrease drops below 20%, which is defined as the lowest absolute decrease with clinical relevance.

#### Data analysis

The principal analysis consists of an intention-to-treat comparison of the number of preoperative severe drainage-related complications. The research hypothesis will be evaluated using a Wilcoxon/Mann–Whitney rank-sum test with a two-sided 0.05 significance level. The comparison will be expressed in terms of a relative risk and 95% confidence intervals, as determined with multinomial logistic regression.

Subsequent analyses are directed at the secondary endpoints. Continuous data will be presented as the mean with standard deviation or median with interquartile range depending on data distribution, and accordingly evaluated with Mann–Whitney U or t-test statistics. Categorical variables will be evaluated using Pearson’s chi-squared test or Fisher’s Exact test as applicable.

Preplanned subgroup analyses will be based on a dichotomized complication rate to retain statistical power (no complication versus 1 or more complications), and the analyses will consist of logistic regression with the subgroup and randomized treatment added as an interaction term. Subgroup analysis will be performed for patients with Bismuth type 4 tumors, and patients with a left-sided FLR.

### Safety

An independent data safety monitoring board (DSMB), consisting of three independent specialists (surgeon, gastroenterologist, clinical epidemiologist) will evaluate the progress of the trial and examine the unblinded safety variables (serious adverse events, patients completing study follow-up) after 50% of inclusion (53 patients). The DSMB will assist and advise the principal investigators so as to protect the validity and credibility of the trial. Furthermore, annual unblinded safety reports, including all serious adverse events per group, are provided to the institutional review board.

## Discussion

Preoperative biliary drainage in PHC is a technically difficult procedure, associated with a high risk of severe drainage-related complications that may deteriorate the patients’ condition or increase the risk of postoperative morbidity and mortality after liver surgery. The majority of patients referred for surgical treatment have already undergone EBD procedures with attempted stent placement upon referral. However, preoperative EBD in PHC is associated with a high rate of complications: technical failures may occur because proximal tumors at the liver hilum are difficult to cannulate endoscopically; cholangitis may occur because isolated bile ducts with segmental obstruction may be left undrained after contrast injection; and pancreatitis may occur after repeated attempts at contrast injection. As an alternative, PTBD has a high rate of technical success because it offers selective segmental drainage, and it has a theoretically lower incidence of cholangitis because there is no retrograde bacterial contamination from the gut. Percutaneous drains also have the potential advantage of protecting hepaticojejunostomies from leaking postoperatively. Nonetheless, PTBD is associated with other drawbacks, including hemorrhage from perforated liver parenchyma, bile leakage, catheter dislocation, and patient discomfort. The DRAINAGE trial is designed to answer the question if preoperative PTBD is associated with fewer preoperative severe drainage-related complications compared to EBD.

Although PTBD is often in used in Western specialty centers, it has been associated with seeding metastases in 2-5% of patients after resection of PHC [12,19-21]. Consequently, most Eastern specialty centers chose to use preoperative endoscopic nasobiliary drainage (ENBD) as their preferred method, after its efficacy was consolidated in a large series of 164 patients [22]. Nonetheless, Western centers generally decline to use ENBD because it severely impairs patients’ quality of life. ENBD stents drain bile externally through a gastro-duodenal tube, and require bile suppletion either via the oral route or via a second gastro-duodenal tube. Moreover, an association with seeding metastases may not be limited to PTBD, as previous series have also shown seeding metastases after endoscopic drainage methods [23]. The DRAINAGE trial is designed to identify PTBD as the preferred preoperative drainage method despite the reported incidence of seeding metastases, because a negative effect of PTBD on survival after resection of PHC has insufficiently been shown.

The rarity of PHC complicates accrual of a sufficient number of patients in a randomized controlled trial. To overcome this challenge, all centers that specialize in surgical treatment of PHC in the Netherlands participate in the DRAINAGE trial, making it a nationwide study. The chosen “all-comers” design, which allows patients to be included in the study even if they underwent previous inadequate drainage procedures in regional centers before referral, will also improve the patient accrual. Moreover, this study design provides pragmatic results to answer the question how to treat any patient that presents with potentially resectable PHC and biliary obstruction in the future liver remnant.

## Additional files

Additional file 1: **Antibiotics protocol.** Prophylactic antibiotics protocol among participating centers in DRAINAGE trial. This additional file describes the prophylactic antibiotics protocol in all centers that participate in the DRAINAGE trial. Additional file 2: **Case record form DRAINAGE Trial.** This additional file is the standardized case record form (CRF) of the DRAINAGE trial which is used to collect all baseline and outcome data by designated data managers.

## Abbreviations

PHC: Perihilar cholangiocarcinoma
EBD: Endoscopic biliary drainage
PTBD: Percutaneous transhepatic biliary drainage
FLR: Future liver remnant
MDT: Multidisciplinary team meeting
DSMB: Data safety monitoring board

## References

[1] Edge SB (2010). AJCC cancer staging manual.

[2] Popescu I, Dumitrascu T (2014). Curative-intent surgery for hilar cholangiocarcinoma: Prognostic factors for clinical decision making. Langenbecks Arch Surg.

[3] Nuzzo G, Giuliante F, Ardito F, Giovannini I, Aldrighetti L, Belli G (2012). Improvement in perioperative and long-term outcome after surgical treatment of hilar cholangiocarcinoma: Results of an italian multicenter analysis of 440 patients. Arch Surg.

[4] Kaiser GM, Paul A, Sgourakis G, Molmenti EP, Dechêne A, Trarbach T (2013). Novel prognostic scoring system after surgery for klatskin tumor. Am Surg.

[5] Iacono C, Ruzzenente A, Campagnaro T, Bortolasi L, Valdegamberi A, Guglielmi A (2013). Role of preoperative biliary drainage in jaundiced patients who are candidates for pancreatoduodenectomy or hepatic resection: Highlights and drawbacks. Ann Surg.

[6] van der Gaag NA, Kloek JJ, de Castro SMM, Busch ORC, van Gulik TM, Gouma DJ (2009). Preoperative biliary drainage in patients with obstructive jaundice: History and current status. J Gastrointest Surg.

[7] Farges O, Regimbeau JM, Fuks D, Le Treut YP, Cherqui D, Bachellier P (2013). Multicentre european study of preoperative biliary drainage for hilar cholangiocarcinoma. Br J Surg.

[8] Sakata J, Shirai Y, Tsuchiya Y, Wakai T, Nomura T, Hatakeyama K (2009). Preoperative cholangitis independently increases in-hospital mortality after combined major hepatic and bile duct resection for hilar cholangiocarcinoma. Langenbecks Arch Surg.

[9] Nagino M, Ebata T, Yokoyama Y, Igami T, Sugawara G, Takahashi Y (2013). Evolution of surgical treatment for perihilar cholangiocarcinoma: A single-center 34-year review of 574 consecutive resections. Ann Surg.

[10] Yokoyama Y, Ebata T, Igami T, Sugawara G, Mizuno T, Nagino M (2014). The adverse effects of preoperative cholangitis on the outcome of portal vein embolization and subsequent major hepatectomies. Surgery.

[11] Kloek JJ, van der Gaag NA, Aziz Y, Rauws EAJ, van Delden OM, Lameris JS (2010). Endoscopic and percutaneous preoperative biliary drainage in patients with suspected hilar cholangiocarcinoma. J Gastrointest Surg.

[12] Kawakami H, Kuwatani M, Onodera M, Haba S, Eto K, Ehira N (2011). Endoscopic nasobiliary drainage is the most suitable preoperative biliary drainage method in the management of patients with hilar cholangiocarcinoma. J Gastroenterol.

[13] van der Gaag NA, Rauws EAJ, van Eijck CHJ, Bruno MJ, van der Harst E, Kubben FJGM (2010). Preoperative biliary drainage for cancer of the head of the pancreas. N Engl J Med.

[14] Rahbari NN, Garden OJ, Padbury R, Brooke-Smith M, Crawford M, Adam R (2011). Posthepatectomy liver failure: A definition and grading by the international study group of liver surgery (ISGLS). Surgery.

[15] Koch M, Garden OJ, Padbury R, Rahbari NN, Adam R, Capussotti L (2011). Bile leakage after hepatobiliary and pancreatic surgery: A definition and grading of severity by the international study group of liver surgery. Surgery.

[16] Rahbari NN, Garden OJ, Padbury R, Maddern G, Koch M, Hugh TJ (2011). Post-hepatectomy haemorrhage: A definition and grading by the international study group of liver surgery (ISGLS). HPB (Oxford).

[17] Dumonceau J-M, Tringali A, Blero D, Devière J, Laugiers R, Heresbach D (2012). Biliary stenting: Indications, choice of stents and results: European society of gastrointestinal endoscopy (ESGE) clinical guideline. Endoscopy.

[18] Elmunzer BJ, Waljee AK, Elta GH, Taylor JR, Fehmi SMA, Higgins PDR (2008). A meta-analysis of rectal nsaids in the prevention of post-ercp pancreatitis. Gut.

[19] Hwang S, Song G-W, Ha T-Y, Lee Y-J, Kim K-H, Ahn C-S (2012). Reappraisal of percutaneous transhepatic biliary drainage tract recurrence after resection of perihilar bile duct cancer. World J Surg.

[20] Kang MJ, Choi Y-S, Jang J-Y, Han IW, Kim S-W (2013). Catheter tract recurrence after percutaneous biliary drainage for hilar cholangiocarcinoma. World J Surg.

[21] Takahashi Y, Nagino M, Nishio H, Ebata T, Igami T, Nimura Y (2010). Percutaneous transhepatic biliary drainage catheter tract recurrence in cholangiocarcinoma. Br J Surg.

[22] Kawashima H, Itoh A, Ohno E, Itoh Y, Ebata T, Nagino M (2013). Preoperative endoscopic nasobiliary drainage in 164 consecutive patients with suspected perihilar cholangiocarcinoma: A retrospective study of efficacy and risk factors related to complications. Ann Surg.

[23] ten Hoopen-Neumann H, Gerhards MF, van Gulik TM, Bosma A, Verbeek PC, Gouma DJ (1999). Occurrence of implantation metastases after resection of klatskin tumors. Dig Surg.

