# Supplementary material for: Preoperative endoscopic versus percutaneous transhepatic biliary drainage in potentially resectable perihilar cholangiocarcinoma (DRAINAGE trial): design and rationale of a randomized controlled trial
Source: BMC Gastroenterol. 2015 Feb 14;15:20. doi: 10.1186/s12876-015-0251-0 (PMC4332425; doi:10.1186/s12876-015-0251-0)
Supplement: Additional file 1: — Antibiotics protocol. Prophylactic antibiotics protocol among participating centers in DRAINAGE trial. This additional file describes the prophylactic antibiotics protocol in all centers that participate in the DRAINAGE trial. [file 12876_2015_251_MOESM1_ESM.pdf]

Profylactic antibiotics protocol among participating centers in DRAINAGE trial

Hospital site no.	Procedural profylactic antibiotics protocol
1	2000 mg ceftriaxone + 5 mg/kg gentamycin
2	2000 mg ceftriaxone
3	1200 mg Augmentin
4	1200 mg Augmentin
5	1200 mg Augmentin
