# Supplementary material for: Preoperative endoscopic versus percutaneous transhepatic biliary drainage in potentially resectable perihilar cholangiocarcinoma (DRAINAGE trial): design and rationale of a randomized controlled trial
Source: BMC Gastroenterol. 2015 Feb 14;15:20. doi: 10.1186/s12876-015-0251-0 (PMC4332425; doi:10.1186/s12876-015-0251-0)
Supplement: Additional file 2: — Case record form DRAINAGE Trial. This additional file is the standardized case record form (CRF) of the DRAINAGE trial which is used to collect all baseline and outcome data by designated data managers. [file 12876_2015_251_MOESM2_ESM.pdf]

ANTONI VAN LEEUWENHOEK NEDERLANDS KANKER INSTITUUT

Drainage
front page

Please return within one month after registration to: Antoni van Leeuwenhoek datacenter, Post Box 90203, 1006 BE Amsterdam

Patient study number

Patient verification code *(mmyyyy of birth date patient)*

Institute

Physician's name

Principle investigator
Dr. T. M. van Gulik, M.D, PhD.
Surgeon
Department of surgery
AMC, Amsterdam
Meibergdreef 9,
1105 AZ Amsterdam
The Netherlands
Tel.: +31-20-566 5572, sein 81 62571
E-mail: t.m.vangulik@amc.nl

Co-investigator
Dr. J. K. Wiggers, MD

Department of surgery
AMC, Amsterdam
Meibergdreef 9
1105 AZ Amsterdam
The Netherlands
Tel.: +31-20-566 5568, sein 81 65573
E-mail: j.k.wiggers@amc.nl

Co-investigator
Drs. R.J.S. Coelen, MD
Department of surgery
AMC, Amsterdam
Meibergdreef 9
1105 AZ Amsterdam
The Netherlands
Tel.: +31-20-566 3995, sein 81 65573
E-mail: r.j.coelen@amc.nl

Datacenter:
Biometrics Department NKI-AVL
PO Box 90203
1006 BE Amsterdam
Tel. +31 20 512 2668

NAME / FUNCTION: .... DATE: .... SIGNATURE: ....

Drainage

Randomization

Protocol version: 3.0 Feb 2014
CRF version: 2.0 Jun 2014

Institute | Patient number | Patient verification code

Please return within one month after registration to: Antoni van Leeuwenhoek datacenter, Post Box 90203, 1006 BE Amsterdam

Institute

Investigator

Name caller

Patient's birth date *(dd/mm/yyyy)*

Gender *(1= male, 2= female)*

Date informed consent signature *(dd/mm/yyyy)*

**INCLUSION CRITERIA**	**YES**	**NO**
Diagnosis or suspicion of pHCCA		
No apparent signs of irresectability on CT scan and/or MRI, and scheduled to undergo a "curative" liver resection (may need additional lymph node biopsies or a diagnostic laparoscopy to further determine resectability)		
Inadequate pre-operative biliary drainage defined as:<br>○ For drainage naïve patients<br>Serum Total Bilirubin (TB) level ≥ 50 µmol/L;<br>○ For drainage non-naïve patients<br>Persistent hyperbilirubinemia or inadequate drainage of the FRL (future remnant liver)		
Signed informed consent		

**EXCLUSION CRITERIA**	**YES**	**NO**
Incomplete recovery from side-effects of any prior stenting attempt		
Signs of active cholangitis, defined as leukocytes ≥ $10*10^9$/L or antibiotic treatment for a suspicion of cholangitis within the past 5 days		

NAME / FUNCTION: .... DATE: .... SIGNATURE: ....

Drainage

Randomization

Institute | Patient number | Patient verification code

Please return within one month after registration to: Antoni van Leeuwenhoek datacenter, Post Box 90203, 1006 BE Amsterdam

	YES	NO
WHO performance score ≥ 3	☐	☐
Any contraindication for major surgery	☐	☐

	YES	NO
Is patient eligible?	☐	☐

Date of registration *(dd/mm/yyyy)* ☐☐/☐☐/☐☐☐☐

Assigned patient study number ☐☐☐☐

STRATIFICATION FACTORS

Tumor progression into the bilateral segmental bile ducts ☐
1= BC type I, II or III or intrahepatic CCA with involvement of the hepatic duct confluence
2= BC type IV or intrahepatic CCA with bilateral involvement of segmental bile ducts

Drainage naivety ☐
1= drainage naïve
2= prior drainage

Centrum of inclusion ☐
1= AMC
2= Erasmus MC
3= MUMC
4= UMCG

Date of treatment allocation *(dd/mm/yyyy)* ☐☐/☐☐/☐☐☐☐

Treatment arm ☐
1= EBD
2= PTBD

NAME / FUNCTION: .... DATE: .... SIGNATURE: ....

Drainage

Baseline

Protocol version: 3.0 Feb 2014
CRF version: 2.0 Jun 2014

Institute | Patient number | Patient verification code

Please return within one month after registration to: Antoni van Leeuwenhoek datacenter, Post Box 90203, 1006 BE Amsterdam

Date of baseline visit *(dd/mm/yyyy)*

Date informed consent signature *(dd/mm/yyyy)*

Assigned treatment arm *(1= EBD, 2= PTBD)*

DEMOGRAPHY

Patient's birth date *(dd/mm/yyyy)*

Gender *(1= male, 2= female)*

SIGNIFICANT MEDICAL HISTORY

Is the patient suffering from, or has he/she ever suffered from significant medical conditions *(0= no, 1= yes)*
(if yes, please record details below)

Diagnosis	Year of first diagnosis	Status *(1= past, 2= latent, 3= active)*
1...		
2. …		
3...		
4...		

PREVIOUS THERAPY

Prior surgery

Did the patient undergo prior liver, pancreas or biliary/galbladder surgery? *(0=no, 1=yes)*
(if yes, please record details below)

	Date procedure *(mm/yyyy)*
1.	
2.	
3. .... ………	

Drainage

Baseline

Protocol version: 3.0 Feb 2014
CRF version: 2.0 Jun 2014

Institute ______ Patient number ______ Patient verification code ______

Please return within one month after registration to: Antoni van Leeuwenhoek datacenter, Post Box 90203, 1006 BE Amsterdam

Prior drainage procedures (at local hospital)

Did the patient undergo drainage procedures before randomization? *(0= no, 1= yes)* ☐

If yes, please specify

Number of drainage procedures *(1-4)* ☐

Type of drainage *(1= ERCP, 2= PTC, 3= both)* ☐

If ERCP, sphincterotomy performed *(0= no, 1= yes)* ☐

Success of stent insertion through stenosis *(0= no, 1= yes)* ☐

Were there procedure related complications *(0= no, 1= yes)* ☐

If yes, specify ☐

1= cholangitis
2= acute cholecystitis
3= acute pancreatitis
4= stent occlusion/dislocation
5= hemorrhage
6= perforation
7= portal vein thrombosis
8= other, specify……………………………………………………………..

Sample date initial blood chemistry (i.e. prior to drainage at local hospital) *(dd/mm/yyyy)* ☐☐☐☐☐☐☐☐

Total Bilirubin *(µmol/l)* ☐☐☐☐

CLINICAL ASSESSMENT

Date assessment *(dd/mm/yyyy)* ☐☐☐☐☐☐☐☐

Height *(cm)* ☐☐☐

Weight *(kg)* ☐☐☐ . ☐

Approximate weight one year ago *(kg)* ☐☐☐ . ☐

WHO Performance status *(0- 4)* ☐

BRUSH

Was brush performed *(0= no, 1= yes)* ☐

If yes, date performed *(dd/mm/yyyy)* ☐☐☐☐☐☐☐☐

Result brush *(1= not malignant, 2= malignant)* ☐

NAME / FUNCTION: ………………………… DATE: ………………… SIGNATURE: …………………

Institute | Patient number | Patient verification code

Please return within one month after registration to: Antoni van Leeuwenhoek datacenter, Post Box 90203, 1006 BE Amsterdam

PREOPERATIVE DIAGNOSTICS

Echo + Doppler performed *(0= no, 1= yes)*

If yes, date performed *(dd/mm/yyyy)*

Multiphasic CT scan *(0= no, 1= yes)*

If yes, date performed *(dd/mm/yyyy)*

MRI/MRCP performed *(0= no, 1= yes)*

If yes, date performed *(dd/mm/yyyy)*

LIVER PARENCHYMA

Cirrhosis *(0= no, 1= yes)*

Atrophy *(0= no, 1= left, 2= right)*

TUMOR CHARACTERISTICS

Bismuth – Corlette classification *(1= I, 2= II, 3= IIIA, 4=IIIB, 5= IV, 6= intrahepatic right, 7= intrahepatic left)*

Tumor size on CT/MRI (RECIST 1.1) *(mm)*

Growth pattern on CT/MRI *(1-3)*
1= mass forming
2= intraductal
3= periductal

SURGICAL PLANNING
Future remnant liver as specified by MDT meeting

Left liver segments *(0= no, 1= yes)*

Right ventral segments *(0= no, 1= yes)*

Right dorsal segments *(0= no, 1= yes)*

DIAGNOSTIC LAPAROSCOPY *(0= no, 1= yes)*

If yes, date admittance *(dd/mm/yyyy)*

Date of operation *(dd/mm/yyyy)*

Date of discharge *(dd/mm/yyyy)*

Result of diagnostic laparoscopy *(1= resectable, 2= irresectable)*

NAME / FUNCTION: .... DATE: .... SIGNATURE: ....

Institute | Patient number | Patient verification code

Please return within one month after registration to: Antoni van Leeuwenhoek datacenter, Post Box 90203, 1006 BE Amsterdam

HEMATOLOGY

	(dd/mm/yyyy)		(dd/mm/yyyy)	
Sample date	__/__/____		__/__/____	
	Value (enter ND if not done)	(specify if other than standard unit)	Value (enter ND if not done)	(specify if other than standard unit)
CRP *(mg/l)*	____ . __	…………………	____ . __	………………
Hemoglobin *(mmol/l)*	____ . __	…………………	____ . __	………………
WBC count *(10^9/l)*	____ . __	…………………	____ . __	………………
ANC *(10^9/l)*	____ . __	…………………	____ . __	………………
APTT *(sec)*	____ . __	…………………	____ . __	………………
PT-INR *(sec)*	____ . __	…………………	____ . __	………………

CHEMISTRY

	(dd/mm/yyyy)		(dd/mm/yyyy)	
Sample date	__/__/____		__/__/____	
	Value (enter ND if not done)	(specify if other than standard unit)	Value (enter ND if not done)	(specify if other than standard unit)
Total Bilirubin* *(µmol/l)*	____ . __	…………………………	____ . __	……………………..

*(*must be value at randomization: this value is the reference value for evaluation of therapeutic success)*

Direct Bilirubin *(µmol/l)*	____ . __	…………………………	____ . __	…………………….
Alk. phosphatase *(U/l)*	____ . __	…………………………	____ . __	……………………..
Y-GT *(U/l)*	____ . __	…………………………	____ . __	……………………..
Creatinine *(µmol/l)*	____ . __	…………………………	____ . __	……………………..
Albumin *(g/l)*	____ . __	…………………………	____ . __	……………………..
Amylase *(U/l)*	____ . __	…………………………	____ . __	……………………..

Drainage

index drainage procedure = iEBD

Protocol version: 3.0 Feb 2014
CRF version: 2.0 Jun 2014

Institute | Patient number | Patient verification code

Please return within one month after index drainage to: Antoni van Leeuwenhoek datacenter, Post Box 90203, 1006 BE Amsterdam

Date of iEBD procedure *(dd/mm/yyyy)*

PROCEDURE CHARACTERISTICS

Standard antibiotics prophylaxis *(0= no, 1= yes)*

Antibiotics given
1= 2000 mg ceftriaxone + 5 mg/kg gentamycin
2= 2000 mg ceftriaxone alone
3= 1200 mg Augmentin
8= other, specify……………………………………………………………………………

Standard post-ERCP NSAID suppository *(100 mg Diclofenac)* *(0= no, 1= yes)*

Sphincterotomy *(0= no, 1= yes, 2= sphincterotomy performed before study)*

Stent placement through stenosis successful *(0= no, 1= yes)*

If no, reason
1= procedure discontinued before stent placement.
2= unable to cannulate obstruction.
8= other, specify……………………………………………………………………………..

Length of procedure *(hh.mm)* .

Stents removed *(0= no, 1= yes)*

If yes, number of stents removed

Side of stent insertion: Left *(0= no, 1= yes)*

Side of stent insertion: Right ventral *(0= no, 1= yes)*

Side of stent insertion: Right dorsal *(0= no, 1= yes)*

Number of stents inserted

Hospitalized *(0= no, 1= yes)*

If yes from *(dd/mm/yyyy)* till *(dd/mm/yyyy)*

Drainage

index drainage procedure = iEBD

Protocol version: 3.0 Feb 2014
CRF version: 2.0 Jun 2014

Institute ______ Patient number ______ Patient verification code ______

Please return within one month after index drainage to: Antoni van Leeuwenhoek datacenter, Post Box 90203, 1006 BE Amsterdam

ADVERSE EVENTS

Were there any drainage related complications *(period: index procedure until re-drainage or explorative laparotomy)* *(0=no, 1=yes)* ☐

Record the worst grade that occurred according to CTC 4.0

Adverse Event	*Grade*	*Relation to procedure* ***(a)***	*SAE* ***(b)***
1. Cholangitis	☐	☐	☐
2. Acute cholecystitis	☐	☐	☐
3. Acute pancreatitis	☐	☐	☐
4. Hemorrhage	☐	☐	☐
Specify location…………………………………			
5. Perforation	☐	☐	☐
Specify location…………………………………			
6. Portal vein thrombosis	☐	☐	☐
7. Dehydration	☐	☐	☐
8. Stent/catheter dysfunction	☐	☐	☐

Adverse Event	*Grade*	*Relation to procedure* ***(a)***	*SAE* ***(b)***
Other, **only use CTCAE 4.0 terminology**			
15. specify……………………………	☐	☐	☐
16. specify……………………………	☐	☐	☐
17. specify……………………………	☐	☐	☐
18. specify……………………………	☐	☐	☐
19. specify……………………………	☐	☐	☐
20. specify……………………………	☐	☐	☐

(a)
1= unrelated
2= unlikely
3= possible
4= probable
5= certain / definite

(b)
0= no
1= yes

INTERVENTIONS AFTER INDEX DRAINAGE PROCEDURE

Percutaneous intervention (other than PTBD) *(0=no, 1=yes)* ☐

If yes, date *(dd/mm/yyyy)* __/__/____

Laparotomy intervention *(not including planned explorative laparotomy)* *(0=no, 1=yes)* ☐

If yes, date *(dd/mm/yyyy)* __/__/____

ICU management *(0=no, 1=yes)* ☐

If yes, date *(dd/mm/yyyy)* __/__/____

Drainage

index drainage procedure = iPTBD

Protocol version: 3.0 Feb 2014
CRF version: 2.0 Jun 2014

Institute | Patient number | Patient verification code

Please return within one month after index drainage to: Antoni van Leeuwenhoek datacenter, Post Box 90203, 1006 BE Amsterdam

Date of iPTBD procedure *(dd/mm/yyyy)*

PROCEDURE CHARACTERISTICS

Standard antibiotics prophylaxis *(0= no, 1= yes)*

Antibiotics given
1= 2000 mg ceftriaxone + 5 mg/kg gentamycin
2= 2000 mg ceftriaxone alone
3= 1200 mg Augmentin
8= other, specify………………………………………………………………………….

Internal drainage through stenosis successful *(0= no, 1= yes)*
(internal + external drainage)

If no, reason
1= procedure discontinued before stent placement.
2= unable to cannulate proximal bile ducts
3= unable to cannulate obstruction.
4= planned two-stage procedure
8= other, specify…………………………………………………………………………..

Length of procedure *(hh.mm)*

Side of catheter insertion: left *(0= no, 1= yes)*

Side of catheter insertion: right ventral *(0= no, 1= yes)*

Side of catheter insertion: right dorsal *(0= no, 1= yes)*

Number of catheters inserted

Hospitalized from *(dd/mm/yyyy)* till *(dd/mm/yyyy)*

NAME / FUNCTION: …………………… DATE: …………………… SIGNATURE: ……………………

Institute | Patient number | Patient verification code

Please return within one month after index drainage to: Antoni van Leeuwenhoek datacenter, Post Box 90203, 1006 BE Amsterdam

ADVERSE EVENTS

Were there any drainage related complications *(period: index procedure until re-drainage or explorative laparotomy)* *(0=no, 1=yes)* ☐

Record the worst grade that occurred according to CTC 4.0

Adverse Event	*Grade*	*Relation to procedure* ***(a)***	*SAE* ***(b)***
1. Cholangitis	☐	☐	☐
2. Acute cholecystitis	☐	☐	☐
3. Acute pancreatitis	☐	☐	☐
4. Hemorrhage	☐	☐	☐
Specify location………………………………….			
5. Perforation	☐	☐	☐
Specify location………………………………….			
6. Portal vein thrombosis	☐	☐	☐
7. Dehydration	☐	☐	☐
8. Stent/catheter dysfunction	☐	☐	☐

(a)
1= unrelated
2= unlikely
3= possible
4= probable
5= certain / definite

(b)
0= No
1= Yes

Adverse Event	*Grade*	*Relation to procedure* ***(a)***	*SAE* ***(b)***
Other, **only use CTCAE 4.0 terminology**			
15. specify……………………………	☐	☐	☐
16. specify……………………………	☐	☐	☐
17. specify……………………………	☐	☐	☐
18. specify……………………………	☐	☐	☐
19. specify……………………………	☐	☐	☐
20. specify……………………………	☐	☐	☐

INTERVENTIONS AFTER INDEX DRAINAGE PROCEDURE

Percutaneous intervention (other than PTBD) *(0=no, 1=yes)* ☐

If yes, date *(dd/mm/yyyy)*

Laparotomy intervention *(not including planned explorative laparotomy)* *(0=no, 1=yes)* ☐

If yes, date *(dd/mm/yyyy)*

ICU management *(0=no, 1=yes)* ☐

If yes, date *(dd/mm/yyyy)*

Endoscopic versus percutaneous biliary drainage in resectable perihilar cholangiocarcinoma:

Drainage

re-drainage procedure = rEBD

Protocol version: 3.0 Feb 2014
CRF version: 2.0 Jun 2014

Institute | Patient number | Patient verification code

Please return within one month after randomization to: Antoni van Leeuwenhoek datacenter, Post Box 90203, 1006 BE Amsterdam

Date of rEBD procedure *(dd/mm/yyyy)*

Indication of drainage procedure
1= technical failure of previous procedure
2= therapeutic failure previous procedure
3= cholangitis
4= stent dysfunction
5= portal vein embolisation (PVE)
8= other, specify……………………………………………………………………………….

PROCEDURE CHARACTERISTICS

Standard antibiotics prophylaxis *(0= no, 1= yes)*

Antibiotic treatment cholangitis *(0= no, 1= yes)*

Antibiotics given
1= 2000 mg ceftriaxone + 5 mg/kg gentamycin
2= 2000 mg ceftriaxone alone
3= 1200 mg Augmentin
8= other, specify…………………………………………………………………………

Standard post-ERCP NSAID suppository *(100 mg Diclofenac)* *(0= no, 1= yes)*

Sphincterotomy *(0= no, 1= yes, 2= sphincterotomy performed in earlier stage)*

Stent placement through stenosis successful *(0= no, 1= yes)*

If no, reason
1= procedure discontinued before stent placement.
2= unable to cannulate obstruction.
8= other, specify…………………………………………………………………………..

Length of procedure *(hh.mm)* .

Stents removed *(0= no, 1= yes)*

If yes, number of stents removed

Side of stent insertion: Left *(0= no, 1= yes)*

Side of stent insertion: Right ventral *(0= no, 1= yes)*

Side of stent insertion: Right dorsal *(0= no, 1= yes)*

Number of stents inserted

Hospitalized *(0= no, 1= yes)*

If yes from *(dd/mm/yyyy)* till *(dd/mm/yyyy)*

NAME / FUNCTION: ………………………… DATE: ………………… SIGNATURE: …………………

Date of rEBD procedure *(dd/mm/yyyy)*

ADVERSE EVENTS

Were there any drainage related complications *(period: re-drainage untill re-drainage or explorative laparotomy)* *(0=no, 1=yes)*
Record the worst grade that occurred according to CTC 4.0

Adverse Event	*Grade*	*Relation to procedure* ***(a)***	*SAE* ***(b)***
1. Cholangitis			
2. Acute cholecystitis			
3. Acute pancreatitis			
4. Hemorrhage			
Specify location…………………………………			
5. Perforation			
Specify location…………………………………			
6. Portal vein thrombosis			
7. Dehydration			
8. Stent/catheter dysfunction			

Adverse Event	*Grade*	*Relation to procedure* ***(a)***	*SAE* ***(b)***
Other, **only use CTCAE 4.0 terminology**			
15. specify……………………………			
16. specify……………………………			
17. specify……………………………			
18. specify……………………………			
19. specify……………………………			
20. specify……………………………			

(a)
1= unrelated
2= unlikely
3= possible
4= probable
5= certain / definite

(b)
0= No
1= Yes

INTERVENTIONS AFTER RE-DRAINAGE PROCEDURE

Percutaneous intervention (other than PTBD) *(0=no, 1=yes)*

If yes, date *(dd/mm/yyyy)*

Laparotomy intervention *(not including planned explorative laparotomy)* *(0=no, 1=yes)*

If yes, date *(dd/mm/yyyy)*

ICU management *(0=no, 1=yes)*

If yes, date *(dd/mm/yyyy)*

NAME / FUNCTION: ……………………………… DATE: ……………………… SIGNATURE: ………………………

Drainage

re-drainage procedure = rPTBD

Protocol version: 3.0 Feb 2014
CRF version: 2.0 Jun 2014

Institute | Patient number | Patient verification code

Please return within one month after randomization to: Antoni van Leeuwenhoek datacenter, Post Box 90203, 1006 BE Amsterdam

Date of rPTBD procedure *(dd/mm/yyyy)*

Indication of drainage procedure
1= technical failure of previous procedure
2= therapeutic failure previous procedure
3= cholangitis
4= stent dysfunction
5= portal vein embolisation (PVE)
8= other, specify…………………………………………………………………………………….

PROCEDURE CHARACTERISTICS

Standard antibiotics prophylaxis *(0= no, 1= yes)*

Antibiotic treatment cholangitis *(0= no, 1= yes)*

Antibiotics given
1= 2000 mg ceftriaxone + 5 mg/kg gentamycin
2= 2000 mg ceftriaxone alone
3= 1200 mg Augmentin
8= other, specify……………………………………………………………………………

Internal drainage through stenosis succesfull *(0= no, 1= yes)*
(internal + external drainage)

If no, reason
1= procedure discontinued before stent placement.
2= unable to cannulate proximal bile ducts
3= unable to cannulate obstruction.
4= planned two-stage procedure
8= other, specify……………………………………………………………………………..

Length of procedure *(hh.mm)*

Catheter replacement: *(0= no, 1= yes)*

Side of catheter replacement: left *(0= no, 1= yes)*

Side of catheter replacement: right ventral *(0= no, 1= yes)*

Side of catheter replacement: right dorsal *(0= no, 1= yes)*

New catheter insertion: *(0= no, 1= yes)*

Side of new catheter insertion: left *(0= no, 1= yes)*

Side of new catheter insertion: right ventral *(0= no, 1= yes)*

Side of new catheter insertion: right dorsal *(0= no, 1= yes)*

Total number of catheters inserted

Hospitalized from *(dd/mm/yyyy)* till *(dd/mm/yyyy)*

NAME / FUNCTION: …………………………………… DATE: ………………… SIGNATURE: ……………………

Drainage

re-drainage procedure = rPTBD

Protocol version: 3.0 Feb 2014
CRF version: 2.0 Jun 2014

Institute | Patient number | Patient verification code

Please return within one month after randomization to: Antoni van Leeuwenhoek datacenter, Post Box 90203, 1006 BE Amsterdam

Date of rPTBD procedure *(dd/mm/yyyy)*

ADVERSE EVENTS

Were there any drainage related complications *(period: re-drainage untill re-drainage or resection)* *(0=no, 1=yes)*
Record the worst grade that occurred according to CTC 4.0

Adverse Event	*Grade*	*Relation to procedure* ***(a)***	*SAE* ***(b)***
1. Cholangitis			
2. Acute cholecystitis			
3. Acute pancreatitis			
4. Hemorrhage			
Specify location…………………………………			
5. Perforation			
Specify location…………………………………			
6. Portal vein thrombosis			
7. Dehydration			
8. Stent/catheter dysfunction			

(a)
1= unrelated
2= unlikely
3= possible
4= probable
5= certain / definite

(b)
0= No
1= Yes

Adverse Event	*Grade*	*Relation to procedure* ***(a)***	*SAE* ***(b)***
Other, **only use CTCAE 4.0 terminology**			
15. specify……………………………			
16. specify……………………………			
17. specify……………………………			
18. specify……………………………			
19. specify……………………………			
20. specify……………………………			

INTERVENTIONS AFTER RE-DRAINAGE PROCEDURE

Percutaneous intervention (other than PTBD) *(0=no, 1=yes)*

If yes, date *(dd/mm/yyyy)*

Laparotomy intervention *(not including planned explorative laparotomy)* *(0=no, 1=yes)*

If yes, date *(dd/mm/yyyy)*

ICU management *(0=no, 1=yes)*

If yes, date *(dd/mm/yyyy)*

NAME / FUNCTION: ………………………………………… DATE: ……………………………… SIGNATURE: ………………………………

Drainage
Evaluation

Protocol version: 3.0 Feb 2014
CRF version: 2.0 Jun 2014

Institute | Patient number | Patient verification code

Please return within one month after laparotomy to: Antoni van Leeuwenhoek datacenter, Post Box 90203, 1006 BE Amsterdam

Evaluation of total bilirubin levels

	Date *dd/mm/yyyy*	result *µmol/l*
Bilirubin prior to referral *(also prior to first drainage elsewhere)*		
Bilirubin at randomization		
Bilirubin 7 days after technical success		
Bilirubin 14 days after technical success		
Bilirubin preoperatively		

Evaluation of therapeutic success *7 days after technical success*

Was 7-day evaluation performed
0=no
1=yes, after iEBD/iPTBD
2=yes, after rEBD/rPTBD

If yes, please specify

Reduced bile ducts in segments FRL on ultrasound *(0= no, 1= yes)*

Was therapeutic success achieved *(0= no, 1= yes)*
(defined as: ≥ 20% decrease relative to reference bilirubin and decreased bile ducts in FRL)

Re-drainage procedures necessary *(0= no, 1= yes)*
If yes, complete re-drainage procedure pages (rEBD or rPTBD)

Cross over to other drainage procedure *(0= no, 1= yes)*

Number of drainage procedures done between randomization and laparotomy

NAME / FUNCTION: .... DATE: .... SIGNATURE: ....

Drainage

Explorative laparotomy

Protocol version: 3.0 Feb 2014
CRF version: 2.0 Jun 2014

Institute | Patient number | Patient verification code

Please return within one month after laparotomy to: Antoni van Leeuwenhoek datacenter, Post Box 90203, 1006 BE Amsterdam

Date index drainage procedure *(dd/mm/yyyy)*

Portal vein embolization *(0= no, 1= yes)*

Number of drainage procedures after randomization

Date of preoperative bilirubin measurement *(dd/mm/yyyy)*

Preoperative bilirubin (max 2 days before surgery) *(µmol/l)* .

Date of explorative laparotomy *(dd/mm/yyyy)*

Operation date as planned *(0= no, 1= yes)*

If no reason of delay
1= rescheduled for clinical reasons (patient's condition did not allow surgery)
2= rescheduled for organizational reasons

Antibiotic prophylaxis *(0= no, 1= yes)*

Details	Administration *1= oral 2= IV*	daily dose *(mg)*	Start date *(dd/mm/yyyy)*	End date *(dd/mm/yyyy)*
1.				
2. …………………………..				
3. …………………………..				

Resection of the tumor performed *(0= no, 1= yes)*

If no, reason irresectability
1= metastases
2= loco regional ingrowth
9= unknown

Palliative procedures during surgery
1= none
2= cholecystectomy
8= other, specify……………………………………………………………………………

If yes, type of liver resection
1= local resection
2= local + hilus resection
3= left sided hepatectomy, specify
1= 3 segments (hemihepatectomy)
2= 4-5 segments (extended hemihepatectomy)
4= right sided hepatectomy, specify
1= 4 segments (hemihepatectomy)
2= 5 segments (extended hemihepatectomy)
8= other, specify……………………………………………………………………………..

NAME / FUNCTION: .... DATE: .... SIGNATURE: ....

Drainage

Explorative laparotomy

Protocol version: 3.0 Feb 2014
CRF version: 2.0 Jun 2014

Institute | Patient number | Patient verification code

Please return within one month after laparotomy to: Antoni van Leeuwenhoek datacenter, Post Box 90203, 1006 BE Amsterdam

Segment 1 (lobus caudatus) resected *(0= no, 1= yes)*

Remnant liver after resection

Left liver segments *(0= no, 1= yes)*

Right ventral segments *(0= no, 1= yes)*

Right dorsal segments *(0= no, 1= yes)*

Portal vein reconstruction *(0= no, 1= yes)*

Number of created hepaticojejunostomies

With a transanastomotic PTBD drain

Without a transanastomotic PTBD drain

Fill out irrespective of resectability

Length of procedure *(hh.mm)* .

Blood loss *(ml)*

Perioperative blood transfusion *(packed cells)*

Pringle manoeuvre *(clamping of the hepatoduodenal ligament)* *(0= no, 1= yes)*

If yes *(1= continuous, 2= intermittent)*

Total clamping time *(min)*

NAME / FUNCTION: .... DATE: .... SIGNATURE: ....

ANTONI VAN LEEUWENHOEK NEDERLANDS KANKER INSTITUUT

Drainage

Explorative laparotomy

Protocol version: 3.0 Feb 2014
CRF version: 2.0 Jun 2014

Institute | Patient number | Patient verification code

Please return within one month after laparotomy to: Antoni van Leeuwenhoek datacenter, Post Box 90203, 1006 BE Amsterdam

INTERVENTIONS AFTER LAPAROTOMY

Percutaneous intervention (other than PTBD) *(0=no, 1=yes)*

If yes, date *(dd/mm/yyyy)*

Re- Laparotomy *(0=no, 1=yes)*

If yes, date *(dd/mm/yyyy)*

ICU management *(0=no, 1=yes)*

If yes, date *(dd/mm/yyyy)*

PATHOLOGY

Histology *(1=benigne disease, 2= malignant disease)*

If malignant

Tumor differentiation grade *(1=well differentiated, 2= poor differentiated)*

Histology *(1=sclerosing, 2= papillary)*

Resection margins *(0= R0, 1= R1, 2= R2)*

TNM STAGING

T *(0= T0, 1= T1, 2= T2, 3= T3, 4= T4, 5= Tis, 9= Tx)*

N *0= N0, 1=N1, 2= N2, 3= N3, 9= Nx*

M *(0= M0,1= M1,9= Mx)*

NAME / FUNCTION: ....
DATE: ....
SIGNATURE: ....

Endoscopic versus percutaneous biliary drainage in resectable perihilar cholangiocarcinoma:

Drainage

final follow up

Protocol version: 3.0 Feb 2014
CRF version: 2.0 Jun 2014

Institute ____ Patient number ____ Patient verification code ____

Please return within one month after final follow up to: Antoni van Leeuwenhoek datacenter, Post Box 90203, 1006 BE Amsterdam

Final follow up to be done 90 days after explorative laparotomy

Date follow up *(dd/mm/yyyy)* __ __ / __ __ / __ __ __ __

CLINICAL ASSESSMENT

Patient status *(1= alive, 2= dead)* ☐

If "dead" (2), please specify cause of death: ☐

1= protocol cancer
2= second malignancy
3= toxicity
4= cardiovascular
8= other, specify……………………………………..

Was patient admitted to (another) hospital for a post-operative complication *(0= no, 1= yes)* ☐

If yes, date admission *(dd/mm/yyyy)* __ __ / __ __ / __ __ __ __

Date discharge *(dd/mm/yyyy)* __ __ / __ __ / __ __ __ __

Indication for re-admission ☐

1= recurrent disease
2= biliary leakage
3= wound infection
4= portal vein thrombosis
5= emergency relaparotomy
6= pneumonia
8= other, specify………………………………………………………………..

INTERVENTIONS

Endoscopic intervention *(0=no, 1=yes)* ☐

If yes, date *(dd/mm/yyyy)* __ __ / __ __ / __ __ __ __

Percutaneous intervention *(0=no, 1=yes)* ☐

If yes, date *(dd/mm/yyyy)* __ __ / __ __ / __ __ __ __

Laparotomy intervention *(0=no, 1=yes)* ☐

If yes, date *(dd/mm/yyyy)* __ __ / __ __ / __ __ __ __

ICU management *(0=no, 1=yes)* ☐

If yes, date *(dd/mm/yyyy)* __ __ / __ __ / __ __ __ __

NAME / FUNCTION: ………………………… DATE: ………………… SIGNATURE: …………………

Drainage
final follow up

Protocol version: 3.0 Feb 2014
CRF version: 2.0 Jun 2014

Institute | Patient number | Patient verification code

Please return within one month after final follow up to: Antoni van Leeuwenhoek datacenter, Post Box 90203, 1006 BE Amsterdam
Final follow up to be done 90 days after explorative laparotomy

ADVERSE EVENTS

Were there any post-operative complications? *(0=no, 1=yes)* ☐
Record the worst grade that occurred according to CTC 4.0

Adverse Event	*Grade*	*Relation to procedure* ***(a)***	*SAE* ***(b)***
1. Cholangitis	☐	☐	☐
2. Acute cholecystitis	☐	☐	☐
3. Acute pancreatitis	☐	☐	☐
4. Hemorrhage	☐	☐	☐
Specify location…………………………………			
5. Perforation	☐	☐	☐
Specify location…………………………………			
6. Portal vein thrombosis	☐	☐	☐
7. Dehydration	☐	☐	☐
8. Stent dysfunction	☐	☐	☐
9. Hepaticojejunostomy (biliary) leakage	☐	☐	☐
10. Intra-abdominal abscess formation	☐	☐	☐

Adverse Event	*Grade*	*Relation to procedure* ***(a)***	*SAE* ***(b)***
11. Wound infection	☐	☐	☐
12. (emergency) (re) laparotomy	☐	☐	☐
13. Pneumonia	☐	☐	☐
14. Postresectional liver failure	☐	☐	☐
Other, **only use CTCAE 4.0 terminology**			
15. specify……………………………	☐	☐	☐
16. specify……………………………	☐	☐	☐
17. specify……………………………	☐	☐	☐
18. specify……………………………	☐	☐	☐
19. specify……………………………	☐	☐	☐
20. specify……………………………	☐	☐	☐

(a)
1= unrelated
2= unlikely
3= possible
4= probable
5= certain / definite

(b)
0= No
1= Yes

Primary endpoint based on reported adverse events verified with local researcher? *(0=no, 1=yes)* ☐

SIGNATURE INVESTIGATOR

I certify that I have reviewed the data on this Case Report Form and that all information is complete and accurate.

Investigator's name	Investigator's signature	Date*(dd/mm/yyyy)*
…………………………………	………………………………	
